# VAS and NRS, Same or Different? Are Visual Analog Scale Values and Numerical Rating Scale Equally Viable Tools for Assessing Patients after Microdiscectomy?

**DOI:** 10.1155/2022/5337483

**Published:** 2022-03-29

**Authors:** Joanna Bielewicz, Beata Daniluk, Piotr Kamieniak

**Affiliations:** ^1^Department of Neurology, Medical University of Lublin, Lublin, Poland; ^2^Institute of Psychology, Marie Curie-Skłodowska University in Lublin, Lublin, Poland; ^3^Department of Neurosurgery, Medical University of Lublin, Lublin, Poland

## Abstract

**Objectives:**

To compare the viability of the numerical rating scale (NRS) and the visual analogue scale (VAS) as a pain assessment tools among a large cohort of patients who underwent microdiscectomy. *Summary of Background Data*. The pain intensity (PI) reduction is a parameter of surgical treatment efficacy. The two most commonly used scales of PI are NRS and VAS. Many studies have shown strong similarities between those two scales, but the direct interchange is difficult.

**Methods:**

Patients, who underwent microdiscectomy, were prospectively enrolled into the study and assessed using VAS and NRS for the back (NRS-B) and the leg (NRS-L), Short Form of McGill Pain Questionnaire (SF-MPQ) included Pain Rating Index (PRI) and Oswestry Disability Index (ODI) 1 day before and 1 month and 3 months after the procedure.

**Results:**

131 patients were included in the study. NRS-L, NRS-B, VAS, and ODI were significantly lower (*p* < 0.001) 1 month after microdiscectomy. NRS-L and NRS-B ratings remained at a similar level while VAS and ODI decreased after 3 months. The rate of decline of PI measured by NRS-L correlated statistically significant (rs = 0.366; *p* < 0.001) with ODI 1 month after surgery. Before surgery, the most significant correlation was found between ODI and NRS-L (rs = 0.494; *p* < 0.001), the lowest with NRS-B (rs = 0.319; *p* < 0.001). 3 months after surgery, there was higher correlations between ODI and VAS (rs = 0.634) than NRS-L (rs = 0.265). PRI correlated significantly (*p* < 0.001) and more stronger with VAS than with NRS-L and NRS-B in every points of assessment.

**Conclusion:**

The results showed that PI measurements by NRS-L/NRS-B and VAS mutually correlate and impair functionality evaluated by ODI (convergent validity) but in different modes (differential validity). NRS and VAS are not parallel scales and assess different aspects of pain. The measurement of NRS-L 1 month after microdiscectomy seems to give quick insight into the effectiveness of the procedure.

## 1. Introduction

Reliable assessment of discectomy results in patients with low back pain (LBP) and lumbar radicular pain (LRP) still remains a challenge. An evaluative endpoint of such treatment is not defined. The evaluation of LBP/LRP treatment effects is still difficult due to insufficient outcome parameters used [1]. In many studies, the reduction of pain intensity (PI) is still considered a parameter of surgical treatment efficacy [2]. Nowadays, the assessment of pain relies on subjective evaluation, due to lack of objective biochemical markers. One applies different scales which mainly evaluate PI. Two such scales numerical rating scale (NRS) and visual analogue scale (VAS) have been used since 1950 [3]. NRS is 11-points (NRS-11) or 101-points (NRS-101) scale which counts the pain and is wildly used in clinical settings because it is easy to administer and score [4]. Conducting of VAS, the patient is asked for visualization of his pain as a point on 10 cm line presented on paper. Although many studies have shown a high correlations between VAS and NRS [5], NRS shows greater compliance and ease of use compared to VAS [4]. The popularity of both scales sometimes causes VAS to be mistaken as NRS and vice versa. That confusion interferes with objective evaluation and comparing the results of research studies.

Functional disability is another value to consider evaluating endpoint surgery outcome. The Oswestry Disability Index (ODI) derived from the self-reported Oswestry Low Back Pain Questionnaire was used to quantify disability for LBP and is considered as a “functional scale.” The patient is asked to assess how the leg and back pain affects nine, daily activities. It is important to remember that the first question concerns measuring PI by the necessity of painkillers usage. This validated questionnaire was first published by Fairbank et al. in *Physiotherapy* in 1980 [6]. The current version was published in the Spine in the year 2000 [7], and it is now registered with the International Consortium for Health Outcomes Measurement as a standard outcome measure [8]. Currently, ODI is used as a functional indicator of the effectiveness of surgical procedures for treating different vertebral column and spine disorders [9]. ODI also assesses the usefulness of anaesthetic techniques applied during surgery [10], and it is a valuable tool for appraising illness perceptions in a group of patients affected by chronic low back pain [11]. An ODI score ≤22 score could be used as a criterion of treatment success of patients with a lumbar spine disorders [12].

The aim of this study was to assess and compare NRS and VAS in the group of patients who underwent discectomy as a treatment of pharmacologically refractory LRP/LBP. Their correlations with ODI and also with Short Form of McGill Pain Questionnaire (SF-MPQ) and Beck Depression Inventory (BDI) can define their properties. The results of our study can be helpful in determining the most accurate and useful tools to monitor and evaluate treatments effects in this group of patients.

## 2. Patients and Methods

### 2.1. Patients

131 (63 female and 68 male) patients with LBP and/or LRP admitted to the Department of Neurosurgery of the Medical University of Lublin were prospectively enrolled into the study. They were qualified to microdiscectomy. All patients received written and verbal information regarding study procedures as well as sign an informed consent. In accordance with binding legislation in this field, the Ethics Committee of the Medical University of Lublin in Poland approved the protocol as well as the details of the informed consent.

The patients mean age ranged from 18 to 76 (*M* = 38.95, SD = 11.23). 80 (61.1%) had a job requiring hard physical activity. Duration of pain was between 1 and 144 months (*M* = 13.49, SD = 21.01) before surgical treatment.

### 2.2. Neurosurgical Procedure

The inclusion criteria for microdiscectomy were as follows: (1) the age of patients between 18 and 80 years, (2) the diagnosis of clinically symptomatic disc herniation (DH), (3) the confirmation of clinical diagnosis by magnetic resonance imaging (MRI), and (4) persistent pain and lack of conservative treatment effects. The exclusion criteria were as follows: (1) previous corticosteroid therapy during three months preceding surgery, (2) previous spine surgery or spinal stenosis, and (3) coexistence of other medical conditions such as rheumatoid diseases, diabetes, cancer, psychiatric disorders, recent surgery for reason other than DH, pregnancy, and alcohol or drug abuse.

All patients were operated on by the same surgeon who used the standard microdiscectomy method on a single DH level. The procedure was carried out under general anaesthesia.

### 2.3. Clinical Assessment

All patients were assessed according to NRS in the back (NRS-B) and the leg (NRS-L) as well as VAS, SF-MPQ, ODI, and BDI, separately. The subjects were evaluated a day before operation and subsequently one and three months following the procedure by the same investigator.

NRS: the patient is asked to indicate the value of his pain on the scale. A 11-point scale was used, with “0” representing “no pain” and “10” representing the “most severe pain imaginable” at the time of assessment.

VAS: it is assessed on a 100 mm horizontal line. The patient is informed that the left end of the scale represents “no pain” and that the right end represents the “most severe pain imaginable.” The patient is then instructed to mark PI currently being experienced on the line.

ODI: the patient is asked to assess how his leg and back pain affects nine activities: personal care, lifting, walking, sitting, standing, sleeping, employment/homemaking, traveling, and social life. The answer for the first question values PI according to the necessity of painkiller intake. Each answer is scored from 0 to 5. Based on the total score, which ranges from 0 to 50, it is possible to evaluate disability for LBP as minimal, moderate, severe, crippling back pain, or disability which makes the patient bed-bound.

SF-MPQ: it consists of the Pain Rating Index (PRI), Present Pain Intensity (PPI), and VAS. For PRI, the patient is asked to describe the sensory and affective qualities of his experience. Descriptors are rated on an intensity scale as 0 = none, 1 = mild, 2 = moderate, or 3 = severe. PRI is the sum of the intensity values of descriptors which characterize pain. PPI is a six steps scale rating PI, from 0 (“no pain”) to 5 (“excruciating pain”).

BDI (Beck Depression Inventory): the patient is asked to answer 21 questions regarding his mood. Every answer is rated from 0 to 4. The presence and intensity of depressive disturbances are evaluated against total score.

### 2.4. Statistical Analysis

Statistical analysis was performed with the use of the IBM SPSS Statistics (Statistical Package for Social Sciences) software for Windows (Version 25.0, Predictive Solutions Sp.z o o., Poland). The mean and standard deviation values for descriptive analysis were provided. Before comparative analyzes were carried out, all data sets had been tested for normal distribution by Kolmogorov–Smirnov test. Statistical differences between nondependent groups were calculated using the nonparametric Mann–Whitney *U* test. Fridman's rank test and Wilcoxon signed-rank test were used to compare the dependent groups. Significance values have been adjusted by Bonferroni correction for multiple tests. Kendall's W coefficient was used to estimate the effect size. The correlation coefficient of Spearman's rho was employed to assess the associations between variables. The level of significance was *α* = 0.05.

## 3. Results

NRS-L, NRS-B, VAS, ODI, PRI, and PPI scores 1 month and 3 months after microdiscectomy: NRS-L, NRS-B, VAS, ODI, PRI, and PPI scores were all significantly lower (*p* < 0.001) 1 month after discectomy. NRS-L, NRS-B, and PPI scores remained stable while ODI (*p* < 0.001), VAS (*p*=0.018), and PRI (*p*=0.016) scores decreased 3 months after surgery. However, the most significant decrease in PI was recorded in NRS-L (*W* = 0.78) (Table 1).

Correlations of NRS-L, NRS-B, VAS, PRI, and PPI scores with ODI score before surgery: significant correlations between every scale of pain assessment results and ODI score were observed. The highest correlation with ODI score was observed with PPI (*r*_*s*_ = 0.527) and NRS-L (*r*_*s*_ = 0.494), the lowest with NRS-B (*r*_*s*_ = 0.319) results. Increased PI correlates with a greater degree of disability.

Correlations of NRS-L, NRS-B, VAS, PRI, and PPI scores with ODI score 1 and 3 months after surgery: 1 and 3 months after surgery significant correlations were still observed between every scale of pain assessment results with ODI score. In the first month of assessment, higher correlations between PRI (*r*_*s*_ = 0.599), PPI (*r*_*s*_ = 0.584), and VAS (*r*_s_ = 0.560) scores and the weaker between NRS-B (*r*_*s*_ = 0.361) and especially NRS-L (*r*_*s*_ = 0.354) with ODI score were observed. 3 months after surgery, the strength of correlations between ODI with PRI (*r*_*s*_ = 0.722), PPI (*r*_*s*_ = 0.742), VAS (*r*_*s*_ = 0.634), and NRS-B (*r*_*s*_ = 0.418) scores increased but with NRS-L (*r*_*s*_ = 0.265) score decreased (Table 2).

Correlations of NRS-L, NRS-B, VAS, ODI, PRI, and PPI scores with age and pain duration before surgery. The age of patients correlates significantly with VAS (*r*_*s*_ = 0.198, *p*=0.024) and ODI (*r*_*s*_ = .249, *p*=0.004). There were no significant correlations between pain scale scores and pain duration. Comparisons of PI between men and women showed no significant differences except that the PI assessed by PPI was significantly higher in the female's group (*z* = −2.083; *p*=0.037).

Evaluation of the rate of decline of PI 1 month after surgery: The change in the results of pain scales before the operation and 1 month after the operation was calculated as the difference between the results of two measurements. The rate of decline of PI measured by NRS-L, NRS-B, VAS, PRI, and PPI correlated significantly with ODI score. The highest such correlation (*r*_*s*_ = 0.366) was found between results of NRS-L and ODI (Table 3).

Mutual correlations between scores of scales of PI assessment: PRI and PPI scores correlated significantly but more strongly with VAS score than with NRS-L and NRS-B scores before (*r*_s_ = 0.525 *p* < 0.001; *r*_*s*_ = 0.573; *p* < 0.001), 1 (*r*_*s*_ = 0.704; *r*_*s*_ = 0.628), and 3 (*r*_*s*_ = 0.745; *r*_*s*_ = 0776) months after surgery (Table 4).

Assessment of depression by BDI and its correlations with NRS-L, NRS-B, VAS, ODI, PRI, and PPI scores before surgery: before surgery, the severity of depressive symptoms in patients ranged from no to severe depression (*M* = 10.57, SD = 6.92). 53% of patients had no signs of depression (BDI < 10). With the remainder, the intensity of depression had a significant and moderate correlation with ODI score (*r*_*s*_ = 0.440, *p* < 0.001), mild with PRI (*r*_*s*_ = 0.251), NRS-L (*r*_*s*_ = 0.210 *p* < 0.016), and PPI (*r*_*s*_ = 0.196, *p* < 0.025) as well as no significant correlation with NRS-B scores and VAS scores (Table 5).

## 4. Discussion

PI is easy to assess with pain rating scales, but it is not so easy to interpret the intricacies of the obtained scores [5]. Interpretation can depend on the defined group of patients, according to the specific disease manifesting with pain. In our study, homogenous group of patients, qualified and underwent microdiscectomy, was assessed. The results showed that PI measurement by NRS-L/NRS-B and VAS mutually and significantly correlate and also impact and impair functionality evaluated by ODI (convergent validity), which was known [5], but in different modes (differential validity), depended on time of assessment. NRS and VAS are not parallel scales and assess different aspects of pain. The direct conversion cannot be made between NRS and VAS what was concluded by other researchers [5]. The patients qualified for microdiscectomy present a mixture of LBP and LRP with prevalence of one in the same individual. LBP and LRP are considered to be of different origins. LBP is mostly nociceptive, and LRP is mostly neuropathic pain (NP). Additionally, the patients with NP are characterized by so-called individual sensory phenotypes [13, 14], a mixture of positive and negative sensory signs. Before surgery, ODI score had the highest correlation with NRS-L score, and the weakest with NRS-B, which indicates that the pain localized in the leg, not in the back, mostly impaired the functional ability of the patient. Effectiveness of microdiscectomy relies mainly on diminishing leg pain according to NRS-L. The rate of decline of NRS-L has the highest correlation with ODI improvement 1 month after surgery. That is why the measurement of NRS-L 1 month after microdiscectomy seems to give quick and favorable insight into the effectiveness of the procedure. However, a stronger correlation between ODI score and NRS-B score was observed at the same time, higher than the correlation ODI with NRS-L. It could suggest that weaker leg pain allows for more severe perception of back pain which started to greatly affect functionality after surgery. The similarity can be found between the results of our study and research of Klieinstueck et al. published in 2011 [15]. The authors claimed that patients with a higher level of back pain preoperatively showed worse outcomes 12 months after decompression surgery for herniated discs [15]. The more prominent the leg pain was at baseline in relation to back pain, the greater the improvement observed in the multidimensional assessment at 12 months' follow-up. We observed a stronger correlation between VAS and ODI after surgery which was higher than correlations between NRS-L/NRS-B and ODI scores. Furthermore, VAS much more strongly correlated with PRI than NRS-L and NRS-B as before and after microdiscectomy. Such a comparison evaluates the differential validity. PRI is a part of SF-MPQ which was developed as an evolution of McGill Pain Questionnaire (MPQ) [16]. They were designed as a quantitative measure of the subjective experience, which is a pain [17]. It gives insight into pain's character, its course and factors which influence the intensity of pain [16]. The patient describes his condition using 2 major groups of concepts: sensory (11) and affective (4). PRI allows one to treat the pain as a complex phenomenon to which detrimental effects are not exclusively compromised to intensity. Strong correlations between VAS and PRI scores show that VAS seems to not only be one-dimensional tool, which was suggested in previous studies [18], but its results also compromise and are affected by other factors, not only PI. Moreover, the differential validity indicates that VAS, but not NRS, assesses severity of pain, based not only on its intensity but also others, sensory and affective factors. VAS is also age-sensitive. It seems to be a multifaceted scale, not only quantitative, but also qualitative. Methodology of the performance of VAS obliged the patient to imagine the pain, which made perceived sensation more concrete but multidimensional. Intensity of depression correlated poorly with NRS-L and not significantly with NRS-B and VAS scores, so mood disturbances should not influence the results of the above comparison. Additionally, VAS is more precise than NRS. Unlike NRS where results are measured in whole numbers, VAS score is shown in millimeters and so seems to be more detailed. This methodology can influence poor reproducibility of NRS and high sensitivity of VAS [5].

We do not observe a significant change in NRS-L and NRS-B score measured 1 and 3 months after surgery. Otherwise, the assessed VAS score is significantly lower in 3 months than that in 1 month after surgery.

The strength of correlation between VAS and ODI increases but between NRS and ODI decreases 3 months after surgery. VAS seems to be a retrospective assessment (patient evaluates pain in recent time) and NRS current (patient evaluates pain in current time).

The choice of pain scales to evaluate the effects of microdiscectomy should depend on time of assessment.

In summary, based on results of our study, VAS evaluates the pain not only in aspects of its intensity but also in its character and its affective perception by an individual as a complex experience in the recent time while NRS assessed the pain in aspects of its intensity in current time.

Because of the complexity of pain, the multidimensional approach to objective assessment is recommended [19]. In 2015, an international group of 22 specialists in several disciplines of spine care proposed a set of metrics to measure and compare outcomes [20]. It included NRS, ODI, and EQ-5D-3L questionnaire for evaluation of quality of life, and questions assessing work status and analgesic use. Recommended follow-up should be performed at 3 months and 5 years after surgery. Based on the above results of the study, VAS is a more adequate pain rating scale than NRS for assessing of PI at proposed time points.

Differences in PI assessment by NRS and VAS indicate prompt necessity of more objective tools for evaluation of pain, e.g., biochemical markers.

Our study is limited by inadequate assessment of quality of life and BMI. Pain is a complex biopsychosocial experience which affects quality of life. We consider that additional data on quality of life and correlation with results of VAS and NRS with BMI (which influences concentration of some substances which are engaged in processes responsible for neuropathic pain phenomena, such as proinflammatory cytokins) could be valuable and enriching for the discussion.

## Figures and Tables

**Table 1:** NRS-L, NRS-B, VAS, ODI, PRI, and PPI scores 1 month before and 1 and 3 months after microdiscectomy.

	Before (a)	1 month after (b)	3 months after (c)	*χ* ^2^ (*p* value)	Effect size Kendall's W	Pair comparisons
NRS-B	3.54 (3.01)	1.17 (1.97)	0.75 (1.51)	100.47 (<0.001)	0.38	a-b (<0.001)
						a-c (<0.001)
						b-c (>0.05)

NRS-L	5.40 (2.68)	0.85 (1.65)	0.45 (1.22)	203.22 (*p* < 0.001)	0.78	a-b (<0.001)
						a-c (<0.001)
						b-c (>0.05)

PRI	16.6 (10.29)	7.10 (7.92)	6.08 (8.06)	137.63 (*p* < 0.001)	0.52	a-b (<0.001)
						a-c (<0.001)
						b-c (0.016)

PPI	2.47 (1.03)	1.21 (0.93)	1.07 (0.99)	123.64 (*p* < 0.001)	0.47	a-b (<0.001)
						a-c (<0.001)
						b-c (>0.05)

VAS	58.50 (24.13)	22.25 (22.50)	16.77 (21.24)	159.34 (*p* < 0.001)	0.61	a-b (<0.001)
						a-c (<0.001)
						b-c (0.018)

ODI	22.56 (8.79)	14.98 (9.62)	10.90 (8.77)	114.88 (<0.001)	0.44	a-b (<0.001)
						a-c (<0.001)
						b-c (<0.001)

**Table 2:** Correlations of NRS-L, NRS-B, VAS, PRI, and PPI scores with ODI score 1 and 3 months after surgery. ^*∗∗*^*p* < 0.01; ^*∗∗∗*^*p* < 0.001.

		NRS-B	NRS-L	PRI	PPI	VAS
Before	ODI	0.319^*∗∗∗*^	0.494^*∗∗∗*^	0.360^*∗∗∗*^	0.527^*∗∗∗*^	0.449^*∗∗∗*^
		<0.001	<.001	<0.001	<0.001	<0.001

1 month after	ODI	0.361^*∗∗∗*^	0.354^*∗∗∗*^	0.599^*∗∗∗*^	0.584^*∗∗∗*^	0.560^*∗∗∗*^
		<0.001	<0.001	<0.001	<0.001	<0.001

3 months after	ODI	0.418^*∗∗*^	0.265^*∗∗*^	0.722^*∗∗*^	0.742^*∗∗*^	0.634^*∗∗*^
		<0.001	0.002	<0.001	<0.001	<0.001

**Table 3:** Correlations of ODI with the rate of decline of NRS-B, NRS-L, PRI, PPI, and VAS 1 month after surgery. ^*∗*^*p* < 0.05; ^*∗∗*^*p* < 0.01; ^*∗∗∗*^*p* < 0.001.

		NRS-B difference	NRS-L difference	PRI difference	PPI difference	VAS difference
*r*_*s*_	ODI	0.242^*∗∗*^	0.366^*∗∗∗*^	0.218^*∗*^	0.213^*∗*^	0.299^*∗∗*^
		0.005	<0.001	0.012	0.014	0.001

**Table 4:** Mutual correlations of NRS-B, NRS-L, PRI, PPI, and VAS at three time points: 0, before surgery; 1, 1 month after surgery; 3, 3 months after surgery. ^*∗*^*p* < 0.05; ^*∗∗*^*p* < 0.01; ^*∗∗∗*^*p* < 0.001.

Spearman's rho		NRS-B	NRS-L	PRI	PPI	VAS
NRS-B	0		0.319^*∗∗∗*^	0.216^*∗*^	0.355^*∗∗∗*^	0.437^*∗∗∗*^
	1		0.426^*∗∗∗*^	0.371^*∗∗∗*^	0.378^*∗∗∗*^	0.414^*∗∗∗*^
	3		0.470^*∗∗*^	0.403^*∗∗*^	0.434^*∗∗*^	0.557^*∗∗*^

NRS-L	0	0.319^*∗∗∗*^		0.414^*∗∗∗*^	0.554^*∗∗∗*^	0.673^*∗∗∗*^
	1	0.426^*∗∗∗*^		0.422^*∗∗∗*^	0.407^*∗∗∗*^	0.456^*∗∗*^
	3	0.470^*∗∗*^		0.293^*∗∗*^	0.288^*∗∗*^	0.351^*∗∗*^

PRI	0	0.216^*∗*^	0.414^*∗∗∗*^		0.376^*∗∗∗*^	0.525^*∗∗∗*^
	1	0.371^*∗∗∗*^	0.422^*∗∗∗*^		0.697^*∗∗∗*^	0.704^*∗∗*^
	3	0.403^*∗∗*^	0.293^*∗∗*^		0.756^*∗∗*^	0.745^*∗∗*^

PPI	0	0.355^*∗∗∗*^	0.554^*∗∗∗*^	0.376^*∗∗∗*^		0.573^*∗∗∗*^
	1	0.378^*∗∗∗*^	0.407^*∗∗∗*^	0.697^*∗∗∗*^		0.628^*∗∗∗*^
	3	0.434^*∗∗*^	0.288^*∗∗*^	0.756^*∗∗*^		0.776^*∗∗*^

VAS	0	0.437^*∗∗∗*^	0.673^*∗∗∗*^	0.525^*∗∗∗*^	0.573^*∗∗∗*^	
	1	0.414^*∗∗∗*^	0.456^*∗∗∗*^	0.704^*∗∗∗*^	0.628^*∗∗∗*^	
	3	0.557^*∗∗*^	0.351^*∗∗*^	0.745^*∗∗*^	0.776^*∗∗*^	

**Table 5:** Correlations of BDI with NRS-L, NRS-B, VAS, ODI, PRI, and PPI scores before surgery. ^*∗*^*p* < 0.05; ^*∗∗*^*p* < 0.01; ^*∗∗∗*^*p* < 0.001.

		NRS_B	NRS_L	ODI	PRI	PPI	VAS
Spearman's rho	BDI	0.154	0.210^*∗*^	0.440^*∗∗∗*^	0.251^*∗∗*^	0.196^*∗*^	0.136
		0.078	0.016	<0.001	0.004	0.025	0.121

## Data Availability

The data used to support the findings of this study are included within the article.
